# Creation of a Multi-Year Pediatric Candidemia Antibiogram in Georgia Identifies Changing Epidemiology and Resistance Trends

**DOI:** 10.1017/ash.2024.293

**Published:** 2024-09-16

**Authors:** Diane Saint-Victor, Mark Gonzalez, Collin Dubick, Matt Linam

**Affiliations:** Children’s Healthcare of Atlanta; Emory University

## Abstract

**Background:** Invasive candidiasis, including candidemia, is a significant cause of morbidity and mortality in medically complex and immunocompromised children. Understanding the epidemiology and antifungal susceptibility patterns of Candida infections could help guide empiric antifungal therapy. **Methods:** This fungal antibiogram was created at a large quaternary children’s health system in Georgia. Blood isolates positive for Candida spp. from 2019 through 2023 were included. The number and percentage of isolates for each Candida spp was recorded by year and then as the combined 5-year total. The Clinical and Laboratory Standards Institute (CLSI) antifungal interpretative criteria were used, and we only included one unique Candida spp isolate per patient. Due to the limited number of isolates, the combined 5 years of isolates were used to create the fungal antibiogram. Data are shown as percent susceptible using CLSI interpretative criteria and number of isolates. **Results:** Between 2019 and 2023 there were 124 unique blood isolates of Candida spp identified. The most common isolates were C. albicans (33%), C. parapsilosis (27%), C. glabrata (14%) and C. tropicalis (11%). Over the 5 years of the study, the percentage of C. albicans isolates decreased from 47% to 21%. The change in epidemiology was not driven by a single Candida species but varied from year to year. For C. albicans, susceptibility was 100% for fluconazole and micafungin. For C. parapsilosis, susceptibility to fluconazole and micafungin was 97% and 94%, respectively. Fluconazole susceptibility was lowest for C. glabrata (88%) and C. krusei (0%). Using CLSI epidemiological cutoff values (ECV) to evaluate the amphotericin B results, none of the isolates had results greater than the CLSI ECVs. Comparing 2019 and 2023, the percentage of Candida blood isolates resistant to fluconazole increased from 5% to 18.5%. **Conclusion:** C. albicans was the most frequently identified cause of candidemia in children, but there was a gradual increase in fungemia caused by other Candida spp. over the past 5 years including Candida with fluconazole resistance. Overall, our findings demonstrate high susceptibility rates to fluconazole and echinocandins in Candida spp. blood isolates. Further research is needed to identify risk factors for antifungal resistant candidemia in pediatric patients.

**Disclosure:** Mark Gonzalez: Honoria for a one time consulation with NaviDx consulting in May of 2022. Honoria from the American Society for Microbiology for writing of a chapter in the Clinical Microbiology Procedures Handbook.

